# Putting Out the Fire: The Relationship of Pharmacogenetics and Proton Pump Inhibitors for the Treatment of Gastroesophageal Reflux Disease

**DOI:** 10.7759/cureus.2687

**Published:** 2018-05-24

**Authors:** Connor L Zheng, Saeed K Alzghari

**Affiliations:** 1 Pharmacotherapy Education and Research Center, University of Texas Health Science Center at San Antonio; 2 Gulfstream Genomics, Gulfstream Diagnostics, Dallas, USA

**Keywords:** proton pump inhibitors, pharmacogenetics, gastroesophageal reflux disease, cyp450, cyp2c19

## Abstract

Proton pump inhibitors (PPIs) are a mainstay treatment for gastroesophageal reflux disease (GERD) and are mainly metabolized by CYP2C19 in the liver. However, several polymorphisms of CYP2C19 exist that affect the metabolism of PPIs. Due to the large variability of PPI pharmacokinetics among the polymorphisms, this has implications in the management of patients with refractory GERD who may be potentially undertreated. Herein, we discuss the role of CYP2C19 and its relation to PPI therapy, particularly in those with GERD.

## Editorial

Heartburn is a symptom commonly experienced by many, but it may develop into gastroesophageal reflux disease (GERD) when heartburn occurs twice weekly or more. GERD is a chronic condition where the reflux of gastric contents into the esophagus causes symptoms (e.g. heartburn) or complications. GERD affects approximately 10–20% of those in Western countries and is becoming progressively prevalent in developing countries [1]. In the United States alone, GERD represents $15–20 billion in health care costs and is the most frequently diagnosed gastrointestinal disorder [2]. A mainstay treatment of GERD is proton pump inhibitor (PPI) therapy. PPIs are superior to H2-receptor antagonists in treating more severe cases of GERD but are subject to more drug-drug interactions. In particular, PPIs are primarily hepatically metabolized by CYP2C19 enzymes.

Polymorphisms in CYP2C19 may increase or decrease metabolism of PPIs, which ultimately affect the treatment of patients with GERD who may have these polymorphisms. A patient who homozygously expresses the CYP2C19*17 allele is an ultra-rapid metabolizer (UM) of CYP2C19 substrates and may be prone to inadequate treatment of GERD due to the PPI being metabolized much faster than usual. The Dutch Pharmacogenetics Working Group (DPWG) developed specific dosing recommendations for PPIs for UMs with insufficient responses: increase by 50–100% with esomeprazole, increase by 200% with lansoprazole, increase by 100–200% with omeprazole, and increase by 400% with pantoprazole [3]. Rabeprazole and dexlansoprazole, however, have no recommendations due to lack of data available for UMs. Increasing the PPI dose may be beneficial for a UM with inadequate symptom relief. Conversely, poor metabolizer (PM) phenotypes expressing any combination of the CYP2C19*2 or *3 loss-of-function alleles have shown to have higher cure rates of GERD due to decreased clearance of the PPI. The DPWG has no recommendations for dose adjustments of PPIs in the PM population.

A recent study investigating CYP2C19 extensive metabolizer (EM) and UM phenotypes in children found an association with the presence of the *CYP2C19 *allele and anti-reflux surgery (ARS) in those with medically refractory GERD [4]. Pediatric manifestations of GERD are often harder to identify as typical symptoms of heartburn and regurgitation are not easily evaluated in younger patients. ARS is among the most common major surgeries in pediatric patients with GERD that have failed pharmacological treatment and have persistent symptoms. The aforementioned study identified children with confirmed GERD who underwent ARS and found that the CYP2C19*1/*17 and *17/*17 genotypes were a significant predictor of ARS compared to the non-ARS controls [4].

With pharmacogenetic testing, determining a patient's CYP2C19 phenotype would aid in finding potential sources of treatment failure of GERD earlier on, leading to fewer complications and increased quality of life [5]. Although the main benefit of utilizing CYP2C19 metabolizer status to tailor PPI therapy appears to be in the UM population who have difficulties managing their GERD, those who are PMs should also be monitored for the increased risk of PPI toxicity.
